# Decoding Plant Ribosomal Proteins: Multitasking Players in Cellular Games

**DOI:** 10.3390/cells14070473

**Published:** 2025-03-21

**Authors:** Dariusz Stępiński

**Affiliations:** Department of Cytophysiology, Institute of Experimental Biology, Faculty of Biology and Environmental Protection, University of Lodz, Pomorska 141/143, 90-236 Lodz, Poland; dariusz.stepinski@biol.uni.lodz.pl; Tel.: +48-42-6354734

**Keywords:** plant cellular ribosomal proteins, ribosomal protein paralogs, ribosome heterogeneity, plant development, stress response

## Abstract

Ribosomal proteins (RPs) were traditionally considered as ribosome building blocks, serving exclusively in ribosome assembly. However, contemporary research highlights their involvement in additional translational roles, as well as diverse non-ribosomal activities. The functional diversity of RPs is further enriched by the presence of 2–7 paralogs per RP family in plants, suggesting that these proteins may perform distinct, specialized functions. The spatiotemporal expression of RP paralogs allows for the assembly of unique ribosomes (ribosome heterogeneity), enabling the selective translation of specific mRNAs, and producing specialized proteins essential for plant functioning. Additionally, RPs that operate independently of ribosomes as free molecules may regulate a wide range of physiological processes. RPs involved in protein biosynthesis within the cytosol, mitochondria, or plastids are encoded by distinct genes, which account for their functional specialization. Notably, RPs associated with plastid or mitochondrial ribosomes, beyond their canonical roles in these organelles, also contribute to overall plant development and functionality, akin to their cytosolic counterparts. This review explores the roles of RPs in different cellular compartments, the presumed molecular mechanisms underlying their functions, and the involvement of other molecular factors that cooperate with RPs in these processes. In addition to the new RP nomenclature introduced in 2022/2023, the old names are also applied.

## 1. Introduction

**Figure 1:**
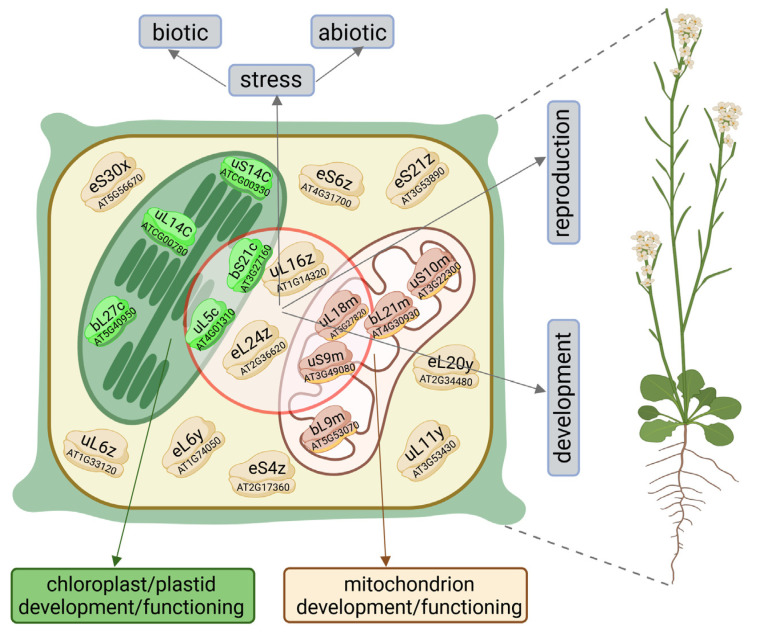
Roles of cellular ribosomal proteins (RPs) in plant functioning. Cytosolic, mitochondrial, and plastid RPs collectively contribute to overall plant development, reproduction, and responses to biotic and abiotic stress factors (the circle encompasses cellular compartments and representative RPs involved in these processes). Additionally, mitochondrial and plastid RPs are specifically involved in the development and functioning of their respective organelles. Selected RPs functioning in *A. thaliana* (discussed also in this review) are depicted in this illustration with their names according to the new nomenclature of RPs, as well as corresponding gene locus IDs [5,6]. Created with BioRender/i71j550.

The functioning of plant cells is coordinated by three genomes: nuclear, mitochondrial, and plastid. Each of these genomes encodes ribosomal proteins (RPs). Respective RPs, in addition to respective ribosomal RNAs (rRNAs), are components of the translational nanomachineries known as ribosomes, which are responsible for translation in the cytosol, mitochondria, and plastids. The RPs of ribosomes functioning in these cellular compartments are encoded by distinct genes (Figure 1). Cytosolic RPs are encoded solely by the nuclear genome, whereas mitochondrial and plastid RPs are encoded by both the nuclear and the genome of the respective organelle. The exact number of both RPs and their corresponding genes is not fully determined in plants; however, in *Arabidopsis thaliana*, 409 genes encoding cytosolic, mitochondrial, and chloroplast RPs, including RP-like proteins, have been identified in the entire genome [1]. All RPs are divided into two groups: those that are a part of the small ribosomal subunits (SSUs; cytosolic 40S, plastid or mitochondrial 30S), referred to as RPSs, and those that constitute the large ribosomal subunits (LSUs; cytosolic 60S, plastid or mitochondrial 50S), referred to as RPLs. It is estimated that SSUs contain between 26 and 40 RPs, while LSUs contain between 41 and 59 RPs [2]. Although the genes encoding RPs undergo dynamic evolution [3], the majority of RP gene sequences are highly conserved among eukaryotes, exhibiting 59–100% nucleotide homology in their coding sequences [4]. Moreover, orthologs of animal RPs are present in *Arabidopsis*, underscoring the structural and functional similarities of eukaryotic ribosomes [2].

RPs exhibit diverse structures, which confer distinct physicochemical properties, including their ability to interact with other proteins or RNAs. As integral components of ribosomal subunits, RPs occupy specific sites that facilitate the connection of SSU with LSU, ensuring the assembly of a fully functional ribosome (80S in eukaryotes or 70S in prokaryotes or organelles). Moreover, RPs play essential roles in translational processes, supporting various stages of translation, e.g., they enable interactions with mRNA during translation initiation [7] or organize binding sites for amino acid-carrying tRNA, as demonstrated in the case of uL16 (formerly known as RPL10) in yeast [8,9].

**Figure 2:**
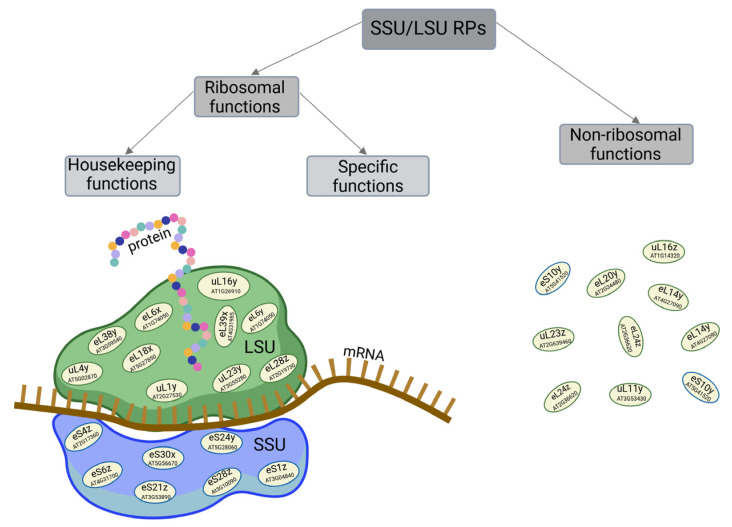
Overview of ribosomal protein (RP) functions. RPs, which form the small (SSU) and large (LSU) ribosomal subunits, are essential for ribosomal functions, primarily the translation of mRNAs encoding both housekeeping proteins and proteins with specialized roles (depicted on the left side of the illustration). Beyond their roles within ribosomes, RPs can also operate independently, performing a variety of non-ribosomal functions (shown on the right side of the illustration). Some cytosolic RPs of SSU and LSU involved in these functions in *A. thaliana*, also discussed in this article, are represented by their updated names and corresponding gene locus IDs [5,6] in this illustration. Created with BioRender/v50y957.

On one hand, RPs perform fundamental housekeeping functions as components of the protein-biosynthesizing machinery, making them indispensable for maintaining cellular homeostasis and, consequently, the proper functioning of the organism (Figure 2). The target of rapamycin (TOR) kinase network plays a central role in the functioning of eukaryotes, including plants, by ensuring optimal cell proliferation, differentiation, growth, and development, even under stress conditions [10]. Ribosome biosynthesis and the production of their core components, such as rRNAs and RPs, are crucial for these processes. This biosynthesis is tightly coordinated and regulated by TOR signaling at the transcriptional, post-transcriptional, and translational levels [11]. Sormani et al. [12] proposed a model for the coordinated control of all cellular RP biosynthesis by three main transcriptional pathways: (1) regulation of the housekeeping needs for cytosolic and mitochondrial ribosomes, mediated by key transcriptional regulators, such as AtTCP20; (2) involvement of a subset of cytosolic RP genes that are coordinately regulated under both non-limiting and stress conditions; and (3) control of ribosome biosynthesis in plastids by regulating both nuclear and plastid genes [12]. TOR-dependent regulation seems to orchestrate various metabolic processes, including those responsible for ribosome component biosynthesis, which occurs in the cytosol, plastids, and mitochondria [13,14]. Cross-talk between TOR and ribosome component biosynthesis is integral to the cellular response to a range of signals, both physiological (including phytohormones, nucleotide, and nutrient availability, such as sulfur or glucose, and energetic status) and external environmental cues (such as circadian rhythm, photoperiod, and biotic and abiotic stresses). This interaction ensures balanced plant growth and development [10,13,14,15]. TOR-mediated reprogramming of the transcriptome, translatome, proteome, and phosphoproteome is accomplished through multiple regulatory axes. Recent studies suggest that optimal homeostasis is achieved by regulating ribosome translational activity, including the modulation of RP functions. In rice (*Oryza sativa*) and *A*. *thaliana* RP activity is influenced by a phosphorylation pathway involving TOR downstream effector, ribosomal protein S6 kinase 1 (S6K1) [16,17]. Phosphorylated by the TORC1-S6K1 pathway, RPS6 is a component of ribosomes translating mRNAs that encode these RPs. Similarly, the translation of specific RP mRNAs is regulated through the TOR-LARP1-5′TOP (TOR-La-related protein 1-5′ track of pyrimidines) signaling axis, which also controls other steps in ribosome biogenesis in *A. thaliana* [14,18]. Additionally, TOR-mediated regulation of RP gene expression at the transcriptional level occurs, for example, through modifications of histones or by regulating the activity of gene promoters, mediated by transcription factors. Furthermore, TOR regulates the activity of the three main RNA polymerases by controlling the binding of transcription factors, also in response to factors like nutrient availability or growth factors [18]. In addition to the mechanism of RP regulation at the main levels—transcriptional and translational—specific expression of RP genes in eukaryotes is largely controlled by changes in the efficiency of RP pre-mRNA splicing and RP mRNA stability [19]. On the other hand, an increasing body of evidence suggests that certain RPs in plants may remodel ribosomes to selectively biosynthesize specific proteins that are critical for processes such as the development of particular organs [20] or the response to various stress stimuli [21,22].

Interestingly, the excess of biosynthesized RPs relative to the demand for ribosome assembly suggests that at least some RPs may be involved in functions other than ribosomal (Figure 2). Indeed, RPs are believed to have more specialized extraribosomal regulatory roles in a variety of processes, either by directly influencing these processes or through interactions with other proteins, including transcriptional factors, or as components of signaling pathways, such as those involving phytohormones [23,24].

Protein biosynthesis is a key process in organism development and is largely dependent on the proper ribosome functioning. Defects in ribosome components can impair cell growth and proliferation, leading to abnormal growth and development. Mutations in the genes encoding RPs not only disrupt the functions of the RPs themselves but also affect the translational properties of ribosomes. A deficiency in even a single RP can destabilize the ribosome and impair its function [22]. Therefore, mutations in the genes of RPs that perform ribosomal functions may result in ribosome insufficiency or dysfunction, which, in turn, leads to impaired overall translation. Damage to an RP-encoding gene, resulting in ribosome dysfunction and impaired translation of specific mRNAs, may cause the loss of specific features. In both cases, the outcome may be phenotypes with abnormal morphology and/or disturbed physiological processes, characterized by either mild defects that are vital or more severe defects that may be lethal [20,25].

Based on mutations of some RP genes in *Arabidopsis*, Lan et al. [5] proposed three groups of RPs whose loss of function results in phenotypes exhibiting common developmental defects of varying severity: subtle developmental phenotypes, severe embryo-lethal phenotypes, and divergent or specialized phenotypes. Mutations in some RPs may lead to common phenotypic abnormalities, while mutations of other RPs produce phenotypes specific to these RPs. The harmfulness of the mutation depends on the functional importance of the given RP. Abnormal phenotypes caused by mutations in RP genes suggest that proteins synthesized by ribosomes with translational RPs or RPs with extraribosomal functions can regulate a variety of plant processes (Figure 1 and Figure 2). However, how RPs influence individual processes and their exact roles in molecular mechanisms and signaling pathways remains largely unknown. In animals, including humans, defects in genes encoding RPs that impair the translational functionality of ribosomes or the extraribosomal functions of RPs may also contribute to the development of numerous diseases, known as ribosomopathies [26].

Studies on *Arabidopsis* have shown that some RPs are essential for proper ribosome function, and their absence can lead to severe developmental disruptions, while other RPs are not required for basic ribosomal activity, allowing the plants to survive without them [27,28]. The essentiality, dispensability, and functions of RPs are determined based on mutations in the genes encoding them. To induce a mutation in such a gene and confirm its effect (or the effect of a natural mutation of RP genes) on the abnormal phenotype in comparison to WT, various genetic tests and manipulations are carried out, including map-based cloning, comparative proteomic analyses [29,30], the use of mutagenic agents (such as ethyl methanesulfonate) to cause point mutations [31], T-DNA insertion into the gene, generation of RNA interference transgenic lines for a specific RP, and complementation assays, i.e., compensating for defects by inserting the correct genes into mutants [32]. Since the effect of the mutation also depends on whether it is a homozygous or heterozygous mutation, and double allele mutants have more severe consequences than single allele knockouts [33], allele tests are also performed. Additionally, the functions of RPs are often assessed based on changes in the expression patterns of the genes encoding them, and genes that are upregulated in specific situations are thought to play essential roles [21,34,35,36,37].

In this review, the functions of RPs are discussed primarily in relation to phenotypes associated with mutations in the genes encoding these RPs and changes in RP gene expression. Although several reviews on the functions of plant RPs have been published, one of them [38] and an excellent research article [5] focus solely on cytosolic RPs or plastid RPs [39], while another addresses mitochondrial RPs [40]. This review aims to expand, complement, and consolidate knowledge on plant cytosolic, plastid, and mitochondrial RPs. It is worth noting that the new nomenclature for RPs proposed by Ban et al. [41], Lan et al. [5], and Scarpin et al. [6], is used in this article, with previously used RP names provided in parentheses.

## 2. Paralogs of RPs and Ribosome Heterogeneity

Studies on the functions of RPs are conducted on a limited number of plant species, primarily those considered model ones, such as maize (*Zea mays*), rice, tobacco (*Nicotiana tabacum*), and most extensively on *A*. *thaliana*. The genome of *A. thaliana* encodes 81 families of cytosolic RPs [42,43]. In plants, genome multiplication has led to the presence of multiple genes within each RP family [5]. Specifically, in *A. thaliana*, each RP family comprises 2–7 members, referred to as paralogs or duplicated RP genes [42]. For example, the uL16 (AtRPL10) family includes three genes: *uL16z* (*AtRPL10A*), *uL16y* (*AtRPL10B*), and *uL16x* (*AtRPL10C*) [23]. Similarly, in rice, a significant proportion of cytosolic RP families have at least 2–3 paralogs, collectively encoded by a total of 255 genes [37]. While paralogs within a family generally share similar structures, they may exhibit slight sequence differences, for instance, in rice, the *eL32* (*OsRPL32*) family genes vary in their 3′ UTR sequences [44]. Overall, paralogs show a similarity of 65–100%, for example, in the cytosolic uL6 (AtRPL9) family of *A. thaliana*, *uL6z* (*AtRPL9B*) and *uL6y* (*AtRPL9C*) are identical, whereas *uL6x* (*AtRPL9D*) shares 89% similarity with them [3,42]. In the yeast *S. cerevisiae*, as in plants, most RP genes are duplicated, which contrasts with mammals, where a given RP type is typically encoded by a single gene [5,6].

In plants, including *A. thaliana*, most RP paralog genes are ubiquitously expressed at the transcriptional level. However, the expression of particular RP genes, even within the same family, can vary significantly. To ensure an adequate pool of ribosomes, the amounts of RPs are regulated in a highly coordinated manner by various mechanisms [11,45], although they may deviate from the ribosome stoichiometry [46]. Consistently, RPs synthesized in excess may not only contribute to ribosome formation but also perform extraribosomal functions [34,38]. It is worth noting that the abundance of RP may not correlate well with transcript levels, and even paralogs may not be expressed at the protein level, despite being transcribed. For example, in the eL6 (AtRPL6) family in *Arabidopsis*, eL6y (AtRPL6B) and eL6x (AtRPL6C) were not detected, while a protein matched eL6z (AtRPL6A) [34]. It is speculated that some ribosome populations might lack certain RPs. These RPs could have specialized extraribosomal functions (Figure 2), with their genes being expressed under specific conditions, such as during particular developmental stages or in response to environmental changes [47,48,49,50], including sucrose supplementation [51].

Some or all paralogs of a given RP family may perform similar functions, providing a sufficient amount of a particular RP to produce an adequate number of primary ribosomes, thereby ensuring overall translation. This is exemplified by *Arabidopsis* uL6 (AtRPL9) members, where, despite variations in their sequence identity, these genes exhibit functional redundancy. While some members within a family may share similar functions, others may have distinct, non-overlapping functions. Consequently, mutations in members of the former group result in similar dose-dependent phenotypes, whereas dysfunctions in the latter group lead to distinct phenotypic outcomes [3]. This indicates that at least some RP paralogs are not functionally equivalent; instead, they play essential and distinct roles within the cell [52,53]. This contrasts with earlier assumptions that the loss of one paralog could be fully compensated by the overexpression of another paralog from the same family [54]. On the other hand, while it is commonly believed that all or certain paralogs within a given RP family perform different functions—as evidenced by the different phenotypes resulting from mutations in the genes encoding them—an alternative perspective is conceivable: these phenotypes could instead arise from variations in the expression patterns of these paralogs rather than from their performing different, specific functions. In this context, it is hypothetically possible that RP paralogs are biochemically equivalent and, as proteins, carry out the same functions, but potentially with varying expression levels, timing, or spatial patterns, which may lead to distinct phenotypic outcomes.

One member of each RP family is incorporated into a given type of ribosome. Combinations of isoforms from different RPs contribute to the formation of diverse ribosomes, a phenomenon known as ribosome heterogeneity. It appears that cells can modulate the expression of given paralogs based on situational needs, producing heterogeneous ribosomes that biosynthesize specific proteins. This ribosome diversity underpins the ribosome filter hypothesis [55], which proposes that specific pools of ribosomes perform specialized translational functions. In this model, unique RPs, acting as translational filters and imposing these ribosomes’ distinct characteristics, are expressed under specific conditions [12,56]. Such specialized ribosomes interpret the ‘ribosome code’, selectively translating specific mRNAs, with the resulting proteins fulfilling unique cellular roles. These roles may be crucial during defined developmental stages, in specific organs, or as adaptive responses to environmental challenges [48,52,57]. This spatiotemporal ribosome heterogeneity likely serves to maintain optimal cellular functioning, supporting proper tissue and organ development and ensuring the organism’s adaptation and survival under both physiological and changing environmental conditions.

Ribosome heterogeneity can be modulated at multiple levels. Beyond the incorporation of different RP paralogs into ribosomes, various post-translational modifications of RPs [2] further enhance ribosome diversity and specificity, shaping the translatome and enabling the translation of specific mRNAs [53]. RP paralogs with such modifications appear to be more prevalent in plants than in other eukaryotes. In addition to canonical RPs, ribosomes contain non-ribosomal proteins that contribute to translation, facilitate ribosome subunit association, or perform other unspecified roles [38,58]. Interestingly, although the turnover rates of most RPs are similar to those of ribosomes (3–5 days), for some RPs or other proteins that enrich ribosomes, the half-lives are much shorter (0.5–1.4 days) [58]. This suggests that the dynamic exchange of those proteins, non-ribosomal proteins with translational roles, or other ribosome-associated factors within ribosomes may act as an additional, rapid regulatory mechanism, further extending the functional specificity of ribosomes in plants. Moreover, the existence of multiple RPs increases the likelihood of mutations within these proteins, which can lead to a wide range of phenotypic defects [48].

## 3. Functions of Cytosolic RPs

**Figure 3:**
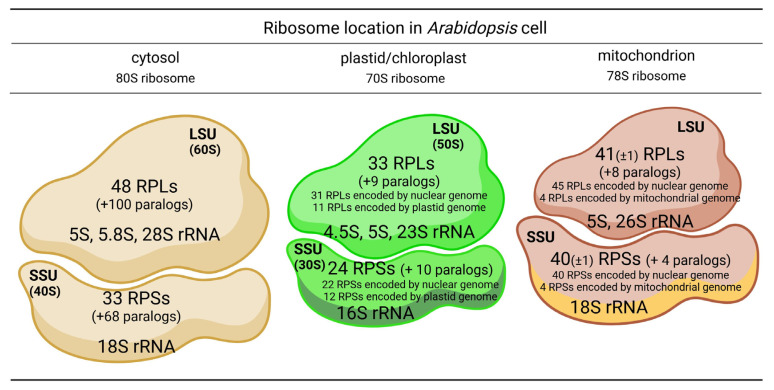
Composition of cytosolic, plastid/chloroplast, and mitochondrial ribosomes in *A. thaliana*. The total number of ribosomal proteins (RPs), including RP paralogs, as well as the number of RPs encoded by the nuclear and organelle genomes, are presented. The rRNA components of each ribosomal subunit are also specified; LSU–large ribosomal subunit, SSU–small ribosomal subunit. Ribosomal data according to literature [6,39,42,61,62]. Created with BioRender/x23t722.

Multiple studies investigating the protein composition of cytosolic ribosomes in *Arabidopsis* have identified similar, though slightly varying, numbers of RPs [59]. Barakat et al. [42] reported the presence of 251 genes encoding cytosolic RPs, including pseudogenes, in this species. However, Hummell et al. [60] later estimated that 242 of these RP genes are functional, excluding pseudogenes. As previously mentioned, these genes are predicted to form 81 families of cytosolic RPs, with each family containing at least two paralogs. Among these, 33 families, encoded by 101 genes, contribute to the formation of SSUs, while 48 families, represented by 148 genes, constitute LSUs (Figure 3). All but one family, P3, which is unique to higher plants, has orthologs in animals [42].

Given that the vast majority of genes encoding paralogs across all cytosolic RP families are expressed at the protein level, it can be inferred that they may perform distinct functions. Indeed, some paralogs serve as structural components of ribosomes, contributing to the formation of diverse ribosome pools. One such pool is dedicated to translating mRNAs encoding housekeeping proteins, while other pools specialize in translating mRNAs coding for proteins with specific functions (Figure 2).

Studies on mutations of various cytosolic RPs have shown that these mutations significantly affect a wide range of developmental processes throughout the plant life cycle, including the development of both vegetative and generative organs. In addition to disrupting physiological processes, the mutated phenotypes exhibit morphological changes in organs and overall plant architecture compared to the wild type (WT). Furthermore, mutations in cytosolic RP genes have revealed that some RPs play roles in responses to biotic and abiotic stress factors (Figure 1), influencing adaptation to changing environmental conditions and contributing to plant defense and immunity. The functions of individual RPs are often inferred from changes in their expression under specific conditions. However, it is important to note that a given RP is frequently one of many components within pathways regulating these processes. Moreover, the involvement of RP in these pathways often depends on many other molecular factors that share a functional relationship. Additionally, studies have highlighted the involvement of RPs in numerous processes regulated by phytohormones.

### 3.1. Cytosolic RPs in Generative Reproduction and Embryonic Development

Some RPs are crucial for the proper progression of processes involved in plant sexual reproduction and embryogenesis at various stages, including gametophyte development, fertilization, and seed development, encompassing the formation of the embryo.

*eL14y* (*AtRPL14B*) appears to play a distinct regulatory role in fertilization and embryogenesis in *Arabidopsis*, despite being expressed in most tissues and organs. Proper growth of the pollen tube, including accurate guidance toward the embryo sac, is essential for successful fertilization. A mutation in the gene encoding eL14y (AtRPL14B) disrupts both male and female gametophyte functions, leading to smaller pollen grains and a marked reduction in pollen tube competitiveness, as well as impairing the pollen tube attraction mechanism. Furthermore, mutations in this gene result in embryo lethality. The severity of these defects varies depending on whether the mutation is homozygous or heterozygous [63].

Similarly, *uL15* (*AtRPL27a*) seems to be essential for proper male and female gametophyte development in *Arabidopsis*, as a mutation in this gene causes defects in pollen grains and embryo sacs, and can even lead to gametophytic lethality due to inhibited mitotic divisions during microgametogenesis and megagametogenesis. Analogous effects are observed in a mutation in the gene encoding karyopherin/importin β, KETCH1, which is responsible for transporting uL15 (AtRPL27a) from the cytoplasm to the cell nucleus. A mutation in either of these genes, resulting in a deficiency of uL15 (AtRPL27a) in the nucleus, impairs ribosome biosynthesis and, consequently, translation. This, in turn, activates pathways that arrest mitotic progression in developing gametophytes [64].

The protein product of the *eL20y* (*AtRPL18aB*) gene is thought to play an important role in controlling early embryo formation in *Arabidopsis*, particularly in regulating cell divisions and determining cell fate during the early stages of embryo development. Irregular cell divisions in very early embryos with the *el20y* (at*rpl18ab*) mutation result in deformation and developmental inhibition at the globular stage, ultimately arresting seed development. This abnormal embryo development may stem from disrupted auxin distribution, which has been observed in the cells of *el20y* (*atrpl18ab*) embryos. Additionally, the *eL20y* (*AtRPL18aB*) gene appears to have a significant role in the functioning of the male gametophyte, as in mutated plants, the competitiveness of pollen grains for growth within the style is markedly reduced, although the *el20y* (*atrpl18ab*) pollen grains can still germinate. In contrast, female gametophytes are only slightly affected [65]. Moreover, *eL20y* (*AtRPL18aB*) is crucial for the proper development and maintenance of suspensor identity during early embryogenesis in *A. thaliana*. The *el20y* (*atrpl18ab*) mutation causes defects in suspensor development, such as excessive proliferation that leads to an abnormal multicellular suspensor. Unlike in WT, where suspensor cell divisions are limited and the structure undergoes programmed cell death (PCD) after the embryo heart stage, suspensors in mutants fail to undergo PCD. Furthermore, the expression of the suspensor marker WOX8 is absent, and auxin accumulation is observed in all suspensor cells in *el20y* (*atrpl18ab*) proembryos, while in WT, auxins are predominantly localized around the hypophysis [66].

### 3.2. Cytosolic RPs in Responses to Stresses

*Abiotic stress*. Under normal conditions, RP genes are differentially expressed depending on the tissue, organ, or developmental stage [35,46]. Moreover, in plants such as *A. thaliana* and *Nicotiana benthamiana*, these genes can also be differentially expressed in response to various stress factors [22]. Changes in the expression of specific RPs may indicate their involvement in plant functioning under specific conditions. For instance, the *AT2G36620* gene, which encodes eL24z (AtRPL24A) in *A. thaliana*, is particularly responsive to osmotic stress. It has been suggested that this gene positively regulates the production of proline, a protein with protective functions during stresses, thereby promoting post-germination development under osmotic stress [67]. However, elevated levels of certain RPs do not necessarily indicate their specific role under particular conditions. It is important to note that the overall proteome, including the riboproteome, changes in response to developmental, environmental, or stress-related factors. Moreover, the levels of most, if not all, RPs and their paralogs are coordinately regulated to maintain optimal ribosome pools, including diversity of ribosomes, under specific conditions.

Differential expression of RP genes at the transcript and protein levels is frequently observed in specific situations, including those influenced by stress factors. The lack of correlation between the translation efficiency of certain mRNAs and the concentration of these mRNAs results from gene-specific translation regulation, as plants can modulate translation under various conditions. Numerous mechanisms control the translation of different mRNAs, including those encoding RPs, thereby influencing ribosome biosynthesis, even under altered conditions. For example, the characteristics of mRNAs provide various opportunities for translation regulation through specific sequences or secondary structures, such as hairpin loops. Among *cis*-elements, specific regulatory sequences within mRNAs—particularly upstream open-reading frames (uORFs)—are the most common gene-specific elements that determine the protein production efficiency of a given mRNA. In addition, *trans*-factors, such as translation initiation factors (eIFs) and their phosphorylation status, influence the translation process under stress conditions. Notably, even if the transcription of RP genes occurs normally, their translational efficiency, and, thus, the level of a given RP, can be significantly altered by various stresses, often due to impaired loading of RP transcripts onto ribosomes. Detailed mechanisms controlling the translation of mRNAs, including those encoding RPs, are described in an extensive review by [45].

In *Arabidopsis* roots, significantly different levels of various transcripts and their corresponding proteins are detected in plants experiencing phosphate (Pi) or iron (Fe) deficiency compared to those grown under Pi- and Fe-sufficient conditions. Some RPs are upregulated, while others are downregulated in response to Pi or Fe deficiency. However, each stress factor affects the expression of distinct sets of RPs. The lack of correlation between the expression of RPs suggests that specific RPs are involved in responses to Pi deficiency, while others are implicated in responses to Fe deficiency. Moreover, the particularly high expression of certain RPs under specific stress conditions, such as uL11y (AtRPL12B) during Pi deficiency, may indicate their critical role in adapting to these conditions [34]. In fact, the varying levels of RPs, including uL11y (AtRPL12B), as well as likely the entire proteome under Pi or Fe deficiency, result from the cell’s response to environmental changes, enabling precise adjustments in protein biosynthesis to meet current demands. Changes in translation are also observed in response to other nutrient limitations, such as sucrose starvation, where translation is negatively regulated by an uORF-dependent mechanism. In this context, mRNAs encoding RPs are significantly excluded from polysomes. Moreover, under abiotic stresses, the phosphorylation status of eIFs changes, leading to the modulation of translation. Ribosome pausing induced by stress also results in mRNA degradation [45].

Consistent with the notice above, although ultraviolet-B radiation (UV-B) generally reduces protein biosynthesis in plants [68], the expression of numerous proteins, including RPs of both SSU and LSU, such as eL6 (ZmRPL6), uL5 (ZmRPL11), eS4 (ZmRPS4), eS8 (ZmRPS8), or uS19 (ZmRPS15), is upregulated in maize [69]. uL16 (RPL10) is a conserved and ubiquitous RP with multiple functions across various organisms. Plants, including maize and *Arabidopsis*, have several genes that encode members of the uL16 (RPL10) family, which are believed to play essential yet distinct roles in both UV-B stress response and development [48,69]. The expression of these genes is tissue- and species-specific [48,49] and is regulated by UV-B in a dose- and time-dependent manner [47]. In *Arabidopsis*, the expression of the *uL16* (*AtRPL10*) family is upregulated through CKB1, a subunit of casein kinase 2 (CK2). The involvement of *uL16* (*AtRPL10*) in UV-B stress signaling in *Arabidopsis* was suggested by observations of *ckb1* mutants, where several members of the uL16 (AtRPL10) family were downregulated. Conversely, the overexpression of *CKB1* increased the expression levels of uL16 (AtRPL10) under UV-B [36]. This response is thought to be mediated by plant hormones, as CKB1 plays an important function in abscisic acid (ABA) and gibberellic acid (GA) signaling [70].

Similarly, in rice, cytosolic RPS and RPL genes are expressed differentially depending on tissue type, developmental stage, internal or environmental cues, applied phytohormones, and the duration of these stimuli. The altered expression of individual RP genes under particular conditions suggests that they play crucial roles in plant responses to such challenges [21,35]. For instance, uL5 (OsRPL5) and eL24 (OsRPL24a) are expressed across all tissues, whereas uL16 (OsRPL10) and eL29 (OsRPL29) exhibit tissue-specific expression in the endosperm and flowers, respectively [35]. The high constitutive expression of RPLs in most tissues during rice plant growth, excluding flowers, indicates that RPLs primarily influence the development of vegetative organs [21,35,71]. In contrast, most RPSs are overexpressed in roots and shoots when exposed to stress factors such as ABA (mimicking osmotic stress), polyethylene glycol (PEG, mimicking drought stress), NaCl, and H_2_O_2_. Only a small subset of RPSs, including uS12 (OsRPS23) and eS19 (OsRPS19), show low expression levels in response to these stressors [21]. For RPL genes, over 60% are upregulated in response to cold, whereas approximately 75% are downregulated under H_2_O_2_ and heat stress conditions [35]. Interestingly, the promoters of most RPL and RPS genes in rice harbor multiple regulatory cis-elements associated with responses to various abiotic and biotic stresses, as well as phytohormones, and their activation likely helps mitigate the adverse effects of stress. For example, the promoters of genes encoding eL6 (OsRPL6) and uL23z (OsRPL23A) contain regulatory elements linked to responses to water deficiency, salt stress, ABA, and PEG. Furthermore, the insertion of transcriptional enhancers into these genes leads to their significant overexpression under water deficiency and other stresses. Consequently, rice plants with enhanced expression of eL6 (OsRPL6) or uL23z (OsRPL23A) exhibit improved tolerance to water limitation and other adverse conditions, highlighting a potential role of these RPs in abiotic stress responses [21,35,71]. From a biotechnological perspective, the promoters of RP genes represent promising targets for genetic engineering to improve stress tolerance. Such modifications could be applied not only to transgenic rice plants but also to other crop species.

Furthermore, the entire *eL32* (*OsRPL32*) gene family, particularly the *el32z* (*OsRPL32A*, *OsRPL32-8.1*) member, appears to be involved in the responses to various abiotic stresses in rice, as all four members of *eL32* (*OsRPL32*) family are downregulated under salt stress in shoots. Notably, *el32z* (*OsRPL32A*) is significantly more downregulated in the salt-sensitive rice variety compared to the tolerant one. This gene is also responsive to other stress treatments, such as cold, drought, and sucrose. The decreased expression of *eL32* (*OsRPL32*) at the transcriptional level is associated with a reduced ability of transcriptional factors to bind specific regulatory cis-sequences in its promoter region under stress conditions. However, the native expression level of this gene is restored when rice seedlings are returned to normal growth conditions [44].

In soybean (*Glycine max*), a chilling-sensitive plant, three genes encoding RPs, *eS6* (*GmRPS6*), *uS15* (*GmRPS13*), and *eL37* (*GmRPL37*), are induced during exposure to low temperatures (5 °C). Interestingly, the expression of these genes is observed only after the third day of cold exposure, whereas the cold stress protein src1 is highly expressed as early as the first day of cold treatment. It has been suggested that delayed response of RP genes may result from the prolonged adaptation of specific signal transduction pathways during cold acclimation [72]. Furthermore, it cannot be ruled out that upregulation of RP genes enhances ribosome biosynthesis and increases translational efficiency under conditions, such as cold, where metabolic processes are slowed down. Additionally, these RPs may be involved in the biosynthesis of specific cold-responsive proteins.

In turn, in *Arabidopsis*, a large number of ribosome biogenesis genes, including those encoding RPs, particularly uL6 (AtRPL9), are upregulated under chilling conditions via the transcription factor HsfA1d (heat shock factor). This regulation is necessary to maintain overall translation and, consequently, the functioning of the plant under chilling stress. The mutational loss of function of RPs, including uL6 (AtRPL9), whose expression is regulated by HsfA1d, inhibits plant growth under chilling but has no effect at normal temperatures. Since translation is particularly vulnerable to chilling stress, the regulation of ribosome biosynthesis by chilling-responsive ribosome components, such as uL6 (AtRPL9) and other RPs, represents an important mechanism that positively influences plant growth under chilling, independently of cold acclimation [73].

*Biotic stress*. On the one hand, some RPs are essential components of plant immunity pathways that counteract pathogen infections. For example, in cucumber (*Cucumis sativus*), eS21 (CsRPS21) plays a role in defense against cucurbit chlorotic yellows virus (CCYV) through its direct interaction with the viral silencing suppressor P22, a protein that weakens plant’s antiviral defense. In this case, the extraribosomal function of eS21 (CsRPS21) involves inhibiting P22 activity, thereby providing the plant with an effective antiviral response [74].

On the other hand, certain RPs can promote infections caused by viral pathogens. A notable example is the eS6 (NbRPS6) family, which serves as an important pro-viral host factor that enhances viral replication, particularly of Tomato spotted wilt orthotospovirus (TSWV). Infection of *N. benthamiana* plants with TSWV induces characteristic symptoms, such as chlorotic leaves, leaf curling, and growth retardation [75]. In contrast, the majority of *N. benthamiana* plants with silenced genes encoding eS6 (NbRPS6) not only lack these symptoms but also exhibit no viral proteins in systemic leaves following TSWV infection [76]. Interestingly, eS6 (NbRPS6) in *N. benthamiana* plays not only a negative but also a defensive role in response to viral infections. This differential response depends on the type of pathogen and arises from the fact that S6K, which regulates eS6 (NbRPS6) activity during pathogen response, can be recruited by certain viruses for their own benefit. The interaction of S6K with specific viral proteins may alter its function, preventing the activating phosphorylation of eS6 (NbRPS6) [77].

In cotton (*Gossypium hirsutum*), eS6 (GhRPS6) seems to be an essential link in the chain involved in defense responses against *Verticillium dahliae* infections, as following inoculation with this pathogenic fungus, an increase in the phosphorylation of eS6 (GhRPS6) at Ser 237 is observed in resistant cultivar compared to susceptible one. This defense pathway involves pathogen-related signaling molecules crucial for plant immunity and systemic acquired resistance, such as SA (salicylic acid) and JA (jasmonic acid). The knockdown of the *eS6* (*GhRPS6*) gene results in reduced levels of SA, JA, and other resistance-related genes, leading to a weakened defensive response and increased susceptibility to *V. dahliae*. Conversely, the overexpression of the *eS6* (*GhRPS6*) gene in transgenic *A. thaliana* plants enhances resistance to the pathogen [78]. The nuclear localization of eS6 (GhRPS6) suggests that this protein may function as an extraribosomal factor required to bolster cotton resistance to *Verticillim* wilt [78]. Therefore, *eS6* (*GhRPS6*) is likely involved in plant resistance to *V. dahliae* through the modulation of hormonal signaling pathways, with its phosphorylation potentially offering enhanced protective effects.

Similarly, the gene encoding eL18 (GaRPL18), which is also involved in an SA-related mechanism but without methyl jasmonate (MeJA), plays a key role in resistance to *Verticillium* wilt disease caused by *V. dahliae* in another cotton species, *Gossypium arboreum*. Wilt-resistant cotton species with a mutated *eL18* (*GaRPL18*) gene are more sensitive to *V. dahliae* than the control plants, as the former inhibit the production of SA and other immune-responsive compounds and are characterized by abnormally long and large vascular bundle cells, which promotes the spreading of the pathogen. In contrast, WT plants treated with exogenous SA or *A. thaliana* plants transformed to overexpress the *eL18* (*GaRPL18*) gene increase SA levels and upregulate the expression of genes related to the SA resistance signaling pathway. This prevents fungal spread in the plants, thereby enhancing their resistance compared to the WT ecotype [79]. The identification of genes responsible for resistance to *V. dahliae*, including *eS6* (*GhRPS6*) and *eL18* (*GaRPL18*), provides a tool for genetic manipulations aimed at producing resistant plants. This would help control disease spread and ultimately improve crop quality and yield, as plants infected with this pathogen suffer lethal damage, leading to significant losses in cotton cultivation.

In turn, proteins from the uL16 (NbQM/NbRPL10) family participate in the positive regulation of numerous genes related to phytohormone-independent defense against bacterial pathogens and genes associated with translational machinery, including other RPs, during non-host pathogen infection in *N. benthamiana*. *N. benthamiana* and *Arabidopsis* plants with a silenced *uL16* (*Nb/AtRPL10*) gene exhibit impaired resistance to non-host pathogens, such as *Pseudomonas syringae* pv. *tomato* T1 and *P. syringae* pv. *tabaci*, respectively. In these plants, the expression of many defense-related genes and genes encoding other RPs is affected. However, silencing the *uL16* (*NbRPL10*) gene does not impair basal resistance to the host pathogen. In contrast, *Arabidopsis* plants overexpressing *uL16* (*AtRPL10*) show reduced sensitivity to the host pathogen *P. syringae* pv. *tomato* DC3000, suggesting that *uL16* (*AtRPL10*) may also be involved in the host pathogen response. Furthermore, some RPs, including uL14 (AtRPL23), eL30 (AtRPL30), and eS30 (AtRPS30), interact with uL16 (AtRPL10), and mutations in the genes encoding these proteins result in increased pathogenicity. This suggests that these RPs may also play a role in non-host defense pathways in *Arabidopsis*. The altered phenotypes with a mutated *uL16* (AtRPL10) gene are believed not to result from disrupted translation due to ribosome dysfunction but rather from impaired extraribosomal functions of uL16 (AtRPL10). Its extraribosomal function is thought to involve the regulation of defense reactions at the transcriptional level, as the expression of genes encoding transcription factors involved in pathogen defense is affected in plants with the mutated *uL16* (*AtRPL10*) gene [80]. It is conceivable that manipulating the expression of genes encoding RPs involved in the defense response could be a strategy for protecting some crop plants against pathogens.

The potential involvement of several RPs of both LSU and SSU in the insect stress response in rice is suggested. In a rice variety resistant to insect pests, the expression levels of genes encoding eL15 (OsRPL15), OsRPL51 (gene ID: Os03g10930.2), and uS7z (OsRPS5a) in response to the brown planthopper (BPH), as well as the expression levels of eL15 (OsRPL15), el20 (OsRPL18a), uL22 (OsRPL22), eL36 (OsRPL36.2), eL38 (OsRPL38), uS7y (OsRPS5), uS4 (OsRPS9.2), and eS25 (OsRPS25a) in response to gall midge, are significantly higher compared to the susceptible variety, suggesting that these RPs participate in the insect stress response. The expression of many other RP genes is also altered, but to a lesser extent. It is plausible that upregulated RPs, in addition to being components of ribosomes and performing translational roles, may also have extraribosomal functions, such as involvement in signal transduction pathways during stress [37].

It is feasible that the ribosome protein composition may change and undergo specific modifications by RP paralogs that are upregulated or specifically expressed in response to biotic or abiotic stress factors. The specialized ribosomes formed in this way may selectively biosynthesize specific proteins involved in the responses and/or adaptation to altered conditions. Moreover, the upregulation of certain RPs may be due to their extraribosomal-specific functions, which are carried out under stressful conditions (Figure 2). Evidence for the important roles of RPs in defense responses comes from the deregulation of paralogs of some RPSs and RPLs, which can form specialized ribosomes in response to the treatment of *A. thaliana* leaves with isonicotinic acid (INA), a compound that induces the plant’s innate defense similarly to that induced by SA and pathogen infection [81].

Interestingly, in organisms other than plants, such as plant pests, genes encoding RPs essential for their development and life can be targeted to combat these pests, thereby protecting plants. One example is the acid phosphatase ribosomal protein (P0), which is necessary for translation and DNA repair in the soybean pod borer (SPB). The plant expression of interfering dsRNA complementary to SpbP0 mRNA disrupts its function, resulting in high mortality of SPB larvae. Soybean plants expressing SpbP0 dsRNA are more effectively protected against SPB larvae compared to the control plants. Therefore, the expression of transferred SpbP0 dsRNA in transgenic plants offers a strategy for protecting them against pests and improving crop yield [82]. It is conceivable that knocking out RPs essential for the functioning and survival of various pests in plants could help control pest populations and produce crops less susceptible to pests.

In general, identifying resistance-related genes would enable biotechnological manipulations and the production of transgenic plants resistant to pathogens and environmental stressors. Genes encoding certain RPs, which are thought to play a role in responses to biotic and abiotic stresses, could serve as potential engineering tools for transgenic plants that would be better protected against various stress factors.

In addition to genetic manipulations, the exogenous application of hormonal growth regulator derivatives can be used to modify the expression of genes encoding RPs to optimize translation under stress conditions. For example, spraying rice leaves with a potassium salt solution of indole-3-butyric acid (IBAK) resulted in the upregulation of genes encoding certain RPSs and RPLs under salt stress. This may enhance protein biosynthesis, improve cell functioning, and promote better growth and development under stress conditions, including salinity [83].

### 3.3. Cytosolic RPs in Plant Growth and Development

eS1z (OsRPS3A) in rice is encoded by the *NARROW LEAF21* (*NAL21*) gene, which is expressed in all tissues but plays a key role in the normal development and growth of leaves. A mutation in this gene (resulting in the *nal21* phenotype) leads to abnormal leaf blade morphology, defects in the leaf vascular system, and reduced plant height. These abnormalities arise because auxin response factors (ARFs, such as those encoded by *OsARF11* or *OsARF16*), which are crucial for normal leaf development, exhibit an impaired response to auxins, and the transcription factor essential for lateral leaf blade outgrowth (encoded by *WUSCHEL-RELATED HOMEOBOX 3A*, *OsWOX3A*) are repressed at translational level in the *nal21* mutant. As a result, the protein expression levels of these factors are downregulated in *nal21*, even though their mRNA levels remain comparable to those in WT plants. In fact, the *nal21* mutant, due to eS1z (OsRPS3A) deficiency, shows disrupted functioning of SSUs and a significant reduction in the number of free SSUs, which are crucial for the initiation of translation, ultimately lowering the overall translational efficiency. It is proposed that eS1z (OsRPS3A) regulates leaf morphology by controlling the expression of polycistronic auxin-responsive transcription factors that govern leaf development. This regulation occurs through uORFs during translation initiation and reinitiation. Impaired translation in *nal21* phenotypes perturbs the cell cycle, leading to reduced cell proliferation and expansion, which ultimately results in smaller organs and plants [84].

Similarly, several genes encoding RPs of SSU and LSU have been identified as crucial for leaf development in *A. thaliana*. These include eS6z (AtRPS6A), eS21z (AtRPS21B), eS24y (AtRPS24B), eS28y (AtRPS28B), eS28z (AtRPS28A), as well as uL1y (AtRPL10aB), uL4y (AtRPL4D), uL30y (AtRPL7B), eL18x (AtRPL18C), eL28z (AtRPL28A), eL38y (AtRPL38B), and eL39x (AtRPL39C). Mutations in any of these genes lead to abnormal development of these organs due to reduced cell proliferation and size, increased abaxialization of leaves, and disorganization of the palisade mesophyll. The phenotypes with a deficiency of these RPs are believed to be the result of the biogenesis of dysfunctional ribosomes, which, in turn, impairs the translation of specific mRNAs that encode proteins involved in processes critical for normal leaf development [25].

The expression of two isoforms of the uL23 (AtRPL23a) family in *Arabidopsis*, uL23z (AtRPL23aA) and uL23y (AtRPL23aB), is crucial for plant viability, as phenotypes with silencing both genes result in their lethality. While the knockdown of uL23z (AtRPL23aA) alone produces a phenotype characterized by retarded growth and abnormal organ morphology, the knockdown of uL23y (AtRPL23aB) has no observable effects on plant growth and development. This suggests that the two RPs are not functionally equivalent and may serve as components of heterogeneous ribosomes dedicated to distinct physiological processes. The exclusive accumulation of both RPs in the nucleoli further supports their role in ribosome biogenesis. It is hypothesized that ribosome deficiency caused by mutations in either *uL23z* (*AtRPL23aA*) or *uL23y* (*AtRPL23aB*) may lead to reduced biosynthesis of essential protein factors, including those involved in auxin signaling pathways [85]. The defective phenotype observed in the *ul23z* (*atrpl23aa*) mutant likely arises from impaired ribosome biosynthesis, which may result not only from the absence of uL23z (AtRPL23aA) as a protein building block of ribosomes but also from disrupted pre-rRNA processing. This is consistent with the hypothesis that uL23z (AtRPL23aA) plays an additional extraribosomal role, potentially contributing to pre-rRNA processing under physiological conditions [86]. A compelling piece of evidence supporting this extraribosomal function is the high affinity of uL23z (AtRPL23aA) for nucleolin, a feature not observed in uL23y (AtRPL23aB). This interaction enables uL23z (AtRPL23aA) to target the nucleolus more efficiently than uL23y (AtRPL23aB) [85].

uL16z (AtRPL10A), one of the three members of the uL16 (AtRPL10) family in *Arabidopsis*, appears to play an important role in the early stages of plant development, as it is highly expressed in cells of all tissues, particularly in meristematic ones, during seed germination, and the initial phases of seedling growth. Additionally, its expression is upregulated in stomatal cells, indicating its involvement in stomatal function. The regulation of these processes may be mediated by ABA-related signaling pathways through uL16z (AtRPL10A) during the early stages of *Arabidopsis* seedling development. This is supported by observations that early developmental stages of the *ul16z* (at*rpl10a*) mutant exhibit reduced ABA sensitivity, developmental impairments, and decreased ABA-mediated inhibition of stomatal closure compared to WT plants. It is suggested that uL16z (AtRPL10A) may perform extraribosomal functions, including interactions with factors involved in plant morphogenesis, growth, development, and stomatal closure. uL16z (AtRPL10A) may regulate the expression of genes related to these processes at the transcriptional level. In contrast, the other two members of the uL16 (AtRPL10) family, uL16y (AtRPL10B) and uL16x (AtRPL10C), do not exhibit such features [23].

Depending on the RP family, its members may have distinct functions or lack functional specialization altogether. In *Arabidopsis*, the three genes of the cytosolic eS10 (AtRPS10) family do not appear to exhibit unique functional specialization, as a mutation in one member can be compensated by the upregulation of the remaining members. This indicates that these RPs function in a manner dependent on their overall quantitative levels. Under normal conditions, maintaining a specific level of gene expression by each member, referred to as the ‘dose effect’, appears to be critical. For example, the *es10y* (*atrps10b*) mutation results in a phenotype characterized by defects in axillary shoot initiation, including the failure to produce axillary leaf primordia. In this case, the normal expression of the remaining eS10 (AtRPS10) family members does not effectively compensate for the loss of *eS10y* (*AtRPS10B*). Consequently, *eS10y* (*AtRPS10B*) is considered to play a crucial role in shoot meristem function, including shoot branching by promoting axillary shoot development from the early stages of bud formation and potentially throughout subsequent bud growth. These processes are likely regulated by auxins, with eS10y (AtRPS10B) playing a mediating role [87].

### 3.4. Cytosolic RPs in Other Processes

A growing body of research suggests that some RPs may have extraribosomal functions, including the regulation of various processes, such as nuclear activities. One such dual regulatory role appears to be performed by eS6 (AtRPS6) in the regulation of rDNA transcription and, consequently, ribosome biogenesis in *Arabidopsis*. On one hand, the unphosphorylated form of eS6 (AtRPS6) negatively regulates rDNA transcription by forming a complex with, and thereby activating, the plant-specific histone deacetylase (AtHD2B). On the other hand, when eS6 (AtRPS6) is phosphorylated by ribosomal protein S6 kinase 1 (AtS6K1), it interacts with nucleosome assembly protein 1 (AtNAP1). This interaction eliminates the repressive effect of eS6-(AtRPS6)-AtHD2B complex, thereby positively regulating rDNA transcription [88].

In addition, certain RPs are responsible for specific cellular processes. For instance, a clear link has been established between uL4y (AtRPL4D) and the biosynthesis of lipids, particularly sterols, and sphingolipids, which are critical for vacuole trafficking in *Arabidopsis*. uL4y (AtRPL4D) is essential for the translation of proteins involved in the biosynthesis of these lipids. Furthermore, uL4y (AtRPL4D) plays a role in auxin-regulated development in this species. Mutant phenotypes (*ul4y (atrpl4d*)) exhibit translational defects caused by a reduction in polysome-bound mRNAs encoding proteins associated with the lipid metabolism pathway, leading to the downregulation of these proteins. As a result, lipid levels in tissues are diminished, causing defects in vacuole membrane transport and auxin-mediated tissue differentiation [89]. It is likely that uL4y (AtRPL4D) regulates the translation of proteins specific to these processes as part of specialized ribosomes.

The functions of cytosolic RPs discussed in this section are summarized in Table 1.

## 4. Functions of Plastid RPs (PRPs)

Plastids, like mitochondria, evolved by endosymbiosis. Consequently, plastid ribosomes (chlororibosomes) are highly similar to bacterial ribosomes. Plastid RPs (PRPs), which are components of plastid ribosomes, exhibit significant homology to RPs in *E. coli*. However, PRPs in plastids generally have higher molecular weights [91]. Furthermore, part of the bacterial genome was transferred to the nuclear genome during evolution. As a result, PRPs are encoded by both the plastid and nuclear genomes [92]. In *A. thaliana*, among the total of 76 PRPs, including PRP paralogs, uL2Cy (AtPRPL2B), uL2Cz (AtPRPL2A), uL14C (AtPRPL14), uL16C (AtPRPL16), bL20C (AtPRPL20), uL22C (AtPRPL22), uL23Cy (AtPRPL23B), uL23Cz (AtPRPL23A), uL32C (AtPRPL32), bL33C (AtPRPL33), and bL36C (AtPRPL36), as well as uS2C (AtPRPS2), uS3C (AtPRPS3), uS7Cy (AtPRPS7B), uS7Cz (AtPRPS7), uS8C (AtPRPS8), uS11C (AtPRPS11), bS12C (AtPRPS12), uS14C (AtPRPS14), uS15C (AtPRPS15), bS16C (AtPRP16), bS18C (AtPRPS18), and uS19C (AtPRPS19), are encoded by the plastid genome, while the remaining PRPs are encoded by the nuclear genome (Figure 3) [6,33,39,93]. The 70S ribosome of spinach chloroplasts includes 23S, 4.5S, 5S, and 16S rRNAs, along with 58 PRPs. Of these, 33PRPs form the large subunit (50S), with 25 encoded by the nuclear genome and 8 by the plastid genome. The small subunit (30S) comprises 25 PRPs, of which 13 are encoded by the nuclear genome and 12 by the plastid genome. In addition to PRPs, plastid ribosomes are combined with an additional protein known as the ribosome recycling factor [94,95,96].

Plastids are characteristic of and essential to plants, and PRPs play a crucial role in plastid ribosome biogenesis, which is integral to the biosynthesis of plastid genome-encoded proteins. This, in turn, influences plastid gene expression, making PRPs indispensable for cell viability and overall plant development [97]. Furthermore, impairments in plastid translation reduce the expression of photosynthetic genes encoded by the nuclear genome [98,99,100]. Plastid translation is particularly critical during chloroplast differentiation and early stages of chloroplast development, as well as during photosynthesis when translational efficiency peaks [27,101]. Studies on PRPs in various plant species, including *A. thaliana*, rice, and maize, indicate that while PRPs are fundamental to plastid development and function, and mutations in these proteins result in spectacular chloroplast developmental and functional defects manifesting as phenotypes with albino organs [102,103]. PRPs are generally vital for many cellular processes and play significant roles in plant development across various stages, from embryogenesis to later stages, particularly during seedling growth. Deficiencies or absence of specific PRPs can have dramatic effects on plant development [27,39,103].

The importance of individual PRPs varies. Some PRPs are critical for proper photosynthesis, plant development, and survival. For example, tobacco PRPs such as uS2C (NtPRPS2), uS4C (NtPRPS4) bS18C (NtPRPS18), and bL20C (NtPRPL20) are essential. Defects in these proteins result in abnormal chloroplast development, disrupted chlorophyll biosynthesis, and impaired photosynthesis, ultimately affecting plastid functioning and potentially leading to embryo or plant lethality. Conversely, other PRPs, such as bL33C (NtRPL33), are not essential. Knockouts of these PRPs do not cause significant damage and plants can complete their life cycles at least under standard conditions, while under stress, such as low temperature, impaired growth and development may occur, as seen in tobacco plants [27,29,33,39,91,103,104,105]. In addition to being structural components of plastid translational machinery, the functional effects of individual PRPs depend on the molecular roles within the plastid ribosomal complex, including interactions with other factors. Thus, they may influence the biosynthesis of various plastid proteins [106]. Mutations in genes encoding these PRPs can lead to defective protein biosynthesis and diverse phenotypes. Interestingly, homologs of a given PRP in different species may influence distinct processes [103].

Apart from PRP orthologues, six nuclear-encoded, plastid-specific ribosomal proteins (PSRPs), designated PSRP1-6, have been identified in spinach and other higher plants [95]. Although their functions remain enigmatic, some PSPRs are indispensable for proper plant development, while others are redundant, with their knockouts not causing any phenotypic defects. PSRPs are suggested to play regulatory translational and non-translational functions. In *Arabidopsis*, cS23 (AtPSRP3), bTHXc (AtPSRP4), and cL37 (AtPSRP5) are essential for proper chloroplast ribosome assembly and function, as well as for the operation of photosynthetic apparatus. In contrast, the absence of cL38 (AtPSRP6) or cS22 (AtPSRP2) does not impair ribosome functions, translation, or normal plant growth and development, as demonstrated by studies on the *cs22* (at*psrp2*) mutant [91]. While cS22 (AtPSRP2) is not critical for plant growth and development under standard conditions, it negatively regulates seed germination and seedling growth under stressful conditions such as salinity, low temperature, or dehydration. This negative effect of cS22 (AtPSRP2) is evidenced by the fact that *cs22* (*atpsrp2*) mutants exhibit better growth under these stressors, whereas transgenic plants overexpressing cS22 (AtPSRP2) show reduced growth compared to WT plants [107]. PSRP1, a homolog of the cyanobacterial ribosome hibernation factor, was initially proposed to have a significant role in chloroplast ribosome translation [108]. However, recent data indicate that *ZmPSRP1* in maize (orthologous to Arabidopsis AT5G24490) does not play a critical role in chloroplast protein synthesis, as phenotypes with mutated *ZmPSRP1* exhibit normal development. Nonetheless, *ZmPSRP1* may have a function in nutrient stress [109]. It is not surprising that a defining feature of plastid ribosomes is their light-regulated efficiency [94,96], potentially mediated, in part, by PSRPs [110]. However, *ZmPSRP1* appears to be insensitive to light regulation [109]. The roles of cL37 (AtPSRP5) and cS22 (AtPSRP2) are briefly discussed further below.

In addition to typical PSRPs, PSRP-like proteins also contribute to plastid functioning. One such protein is *A*. *thaliana* Chloroplast Ribosome-Associated (CRASS) protein, which localizes to the chloroplast stroma, where it physically binds to SSU, most likely via bS1c (AtPRPS1) and uS5c (AtPRPS5). CRASS plays a role in the biogenesis and stability of chloroplast ribosomes, supporting their translational activity, particularly under stressful conditions. Loss of CRASS activity results in minor defects in plants grown under normal conditions, indicating that the protein is not essential for plant survival under such conditions. However, under stressful conditions, including chilling, photosynthetic activity, chloroplast translation, and overall development are significantly impaired [111].

### 4.1. Plastid RPs in Plastid/Chloroplast and Overall Plant Development

Although intensive investigations of PRPs in several model species―primarily *A. thaliana*, rice, maize, and tobacco―have been ongoing for about two decades, and all chloroplast RPs have been identified, the molecular functions only for some PRPs have been determined, particularly in *A. thaliana*. Interestingly, some PRPs are involved in similar processes across different species, as mutations in various PRPs can produce analogous effects. However, the same PRP orthologs in diverse plant species may serve distinct functions, indicating that certain PRPs perform species-specific roles [27,112]. Furthermore, the functionality of some PRPs depends on their post-translational modifications. Similarly to cytosolic RPs, some PRP families include multiple paralog forms, each with distinct functions [94,96].

PRPs are specific to plants, although homologs are also present in bacteria. These proteins are expressed in most plant tissues and organs, including roots, though expression levels can vary significantly across different tissues [103,113,114]. In general, genes encoding PRPs are predominantly expressed in photosynthesizing tissues, where their protein products accumulate in chloroplasts and play a fundamental role in their development and functioning. As illustrated by the examples below, mutations in genes encoding PRPs result in characteristic phenotypes indicative of disturbed chloroplast functions. These dysfunctions are primarily caused by the absence of specific proteins, stemming from impaired translational machinery, which, in turn, is a consequence of mutations in the genes encoding PRPs.

Romani et al. [27] demonstrated the distinct roles of several PRPs in *A. thaliana*. They showed that bS20c (AtPRPS20), uL1c (AtPRPL1), uL4c (AtPRPL4), bL27c (AtPRPL27), and bL35c (AtPRPL35) are critical for proper ribosome functioning during early embryo development, specifically at the globular stage and the transition to the heart stage. The absence of any of these proteins leads to arrested embryo development before the heart stage [27]. Similarly, uL5c (AtPRPL5) is indispensable for early embryo development in *A. thaliana*. A mutation in the gene encoding this protein results in a lethal phenotype after the globular stage, characterized by the development of abnormal chloroplasts lacking thylakoids. This defect arises from impaired plastid ribosomes, which disrupts plastid translation [106]. In contrast, bL28c (AtPRPL28) is essential for development at the later embryo-to-seedling stages. Furthermore, bS1c (AtPRPS1), uS17c (AtPRPS17), and uL24c (AtPRPL24) are required for plastid photosynthetic functions. Mutations in these genes—*bs1c (atprps1*), *us17c* (*atprps17*), and *ul24c* (*atprpl24*)—result in significant reductions in chlorophyll content and photosynthetic efficiency. Phenotypes carrying these mutations display pale green cotyledons and leaves. In addition, *ul24c* (*atprpl24*) and *bs1c (atprps1*) mutants exhibit severely stunted growth. These phenotypic defects are linked to decreased levels of photosynthetic proteins, which stem from impaired biogenesis of the translational machinery due to reduced levels of rRNA transcripts, as observed in the *us17c* (*atprps17*) and *ul24c* (*atprpl24*) mutants [27]. Similarly, the *hcf60* mutation in the nuclear gene *HIGH CHLOROPHYLL FLUORESCENCE 60*, which encodes chloroplast uS17c (ZmRPS17) in maize, generates a lethal pale-green seedling phenotype [112].

A missense mutation in the gene encoding chloroplast uS5c (AtPRPS5) reveals that this gene performs multiple roles in the physiology of *Arabidopsis*. In WT plants, this PRP positively regulates the processing of 16S rRNA, thereby ensuring the proper function of chloroplast ribosomes and the synthesis of proteins, including those involved in photosynthesis. In contrast, the *us5c* (*atprps5*) mutation impairs ribosome function and translation, leading to defective phenotypes characterized by reduced chlorophyll content, yellow leaves, and stunted plant growth. Moreover, the *us5c* (*atprps5*) mutation triggers a cascade effect, downregulating the expression of other PRPs essential for growth and development [115].

uS9c (AtPRPS9) and cL37 (AtPSRP5) (plastid specific RP 5) are essential for chloroplast development and photosynthesis in *Arabidopsis*. uS9c (AtPRPS9) is expressed in green tissues but not in roots, indicating its critical role in photosynthetic functions. In *us9c* (*atprps9*) mutants, chloroplasts exhibit underdeveloped thylakoid membranes, and the efficiency of light energy utilization is significantly lower compared to WT plants. Additionally, uS9c (AtPRPS9), uS11C (AtPRPS11), and cL37 (AtPSRP5) are necessary for plastid rRNA processing. Mutations in the genes encoding these proteins lead to the over-accumulation of rRNA precursors in plastids, accompanied by a deficiency of mature rRNAs. However, the *cs22* (*atpsrp2*) mutation does not affect plastid rRNA processing. This indicates that only certain PRPs and PSRPs are involved in rRNA processing in *Arabidopsis* plastids. The impaired chloroplast rRNA processing is likely a secondary effect of disrupted protein biosynthesis in mutants with defective genes encoding these proteins. Moreover, *uS9c* (*AtPRPS9*) and *cL37* (*AtPSRP5*) are also required for leaf variegation, as the knockdown of these genes suppresses this process. By contrast, the *cs22* (*atpsrp2*) mutation has no effect on leaf variegation. This suggests that different PRPs or PSRPs perform distinct roles in *Arabidopsis* chloroplasts [116].

Low photosynthetic efficiency 2 (LPE2), a nucleus-encoded protein specifically localized to chloroplasts, is predicted to function as bS21c (AtPRPS21), playing a role in photosynthesis and maintaining the carbon/nitrogen balance in *Arabidopsis*. Mutation of the *LPE2* gene disrupts thylakoid membrane composition, alters chloroplast protein profiles, and perturbs the expression of nuclear genes responsible for regulating the carbon/nitrogen balance, which is critical for proper plant growth and development. Consequently, *lpe2* phenotypes exhibit reduced photosynthetic efficiency, pale green cotyledons, and smaller overall plant size. Furthermore, since the *LPE2* gene is expressed throughout the plant’s development, its absence may lead to a shortened life cycle [117].

Interestingly, the *A. thaliana* phenotype resulting from another mutation in the gene encoding bS21c (AtPRPS21), specifically the *ghs1* mutation caused by T-DNA insertion into the *GHS1* (*GLUCOSE HYPERSENSITIVE 1*, At3g27160) gene, is characterized by increased sensitivity to glucose excess. Under such conditions, these mutants show numerous chloroplast defects. The dysfunction of the photosynthetic apparatus, which adversely affects plant development, is attributed to defects in the plastid translational machinery and protein biosynthesis caused by the absence of bS21c (AtPRPS21). Furthermore, the *ghs1* mutants display reduced fertility, suggesting that bS21c (AtPRPS21) also plays a role during the reproductive phase in *Arabidopsis* [118].

The *AL* (*ALBINO LETHAL*) and *ASL* (*ALBINO SEEDLING LETHALITY*) genes in rice are primarily encoded by the nuclear genome. Although they encode different PRPs—e.g., *AL1* encodes bL12c (OsPRPL12) [119], *ASL1* encodes bS20c (OsPRPS20) [120], *ASL2* encodes bL21c (OsPRPL21) [29], and *ASL4* encodes bS1c (OsPRPS1) [121]—these genes appear to have similar functions based on the comparable phenotypes observed in mutants. These genes are predominantly overexpressed in the photosynthesizing tissues of green organs, with their products targeted to chloroplasts, mainly in the leaves of young seedlings, whereas their expression in roots is low. Consequently, these PRPs play a crucial role in chloroplast development and photosynthesis-related processes during the early stages of seedling growth. Proper chloroplast functioning relies on uninterrupted chloroplast protein biosynthesis, which, in turn, depends on the correct assembly and operation of ribosomes with fully functional PRPs encoded in *AL* and *ASLs*. Mutations in these genes result in defective chloroplast development and albino phenotypes characterized by reduced chlorophyll and carotenoid biosynthesis, as well as shortened lifespans, typically limited to the three- or four-leaf stage. These phenotypic defects arise from significant disruptions in chloroplast translation caused by impaired assembly and reduced accumulation of chloroplast ribosomes [29,119,120,121]. Interestingly, in *A. thaliana*, plants carrying the *asl2* mutation exhibit abnormal chloroplast development but remain viable, though they produce a fraction of aborted seeds [122]. In rice, the expression of *ASL1* [120] and *ASL2* genes [29] is regulated by light. This contrasts with maize [123] and spinach [124], where *ASL2* expression is independent of light. Furthermore, numerous plastid- and nuclear-encoded genes involved in chloroplast biogenesis, chlorophyll biosynthesis, and photosynthesis, including those encoding RPs and RNA polymerases—particularly those responsible for chloroplast rRNA biosynthesis—display differential expression in *asl2* [121] and *asl4* [29] tissues compared to WT plants. Some of these genes are downregulated, while others are upregulated, leading to impaired plastid transcription and affecting ribosome biogenesis.

Many studies suggest that the functions of PRPs extend far beyond their immediate roles within plastids. Mutations in PRP-encoding genes reveal their broader influence on the expression of nuclear genome-encoded products. Since the cellular genome is distributed among the plastid, nucleus, and mitochondrion—and all three genomes are essential for the proper development and functioning of plastids, cells, and the entire plants—mechanisms must exist to precisely orchestrate gene expression across these compartments (Figure 1). The communication of signals from the plastid to the nucleus to adjust nuclear gene expression is referred to as retrograde signaling. This process is a common and crucial regulatory mechanism involved in plant physiology, growth, development, and responses to environmental stresses [125]. PRPs may serve as direct or indirect mediators of signal transduction between plastids and the nucleus. The absence of disrupted chloroplast–nuclear signaling in the *al1* mutant suggests that *AL1* is not involved in retrograde signaling [119]. In contrast, the *asl1* mutation affects the expression of both plastid and nuclear genes associated with chlorophyll biosynthesis and chloroplast development [120]. This finding implies that bS20c (OsPRPS20) may participate in the chloroplast–nuclear signaling feedback loop in rice. In *A. thaliana*, the GUNI protein is known to play a role in retrograde signaling to maintain protein homeostasis in chloroplasts. Among the proteins regulated by GUN1 is bS1c (AtPRPS1). When protein homeostasis is disrupted, GUNI integrates signals from the impaired plastids and transmits them to the nuclear control center, triggering adjustments in nuclear gene expression to adapt to the prevailing conditions [126].

The uL18c (At/OsPRPL18) protein, encoded by a nuclear gene in *A. thaliana* and rice, is a conserved protein in plants but exhibits diverse functions across species, with mutations leading to varying phenotypic consequences. The *uL18c* (*At/OsPRPL18*) gene is highly expressed in green tissues, and its protein products have been localized to chloroplasts. It plays an important role in chloroplast development and functions, including chlorophyll biosynthesis. In *A*. *thaliana*, mutation in the gene encoding uL18c (AtPRPL18) results in the arrest of embryo development at the globular stage, producing albino seeds and highlighting its essential role in embryo and seed development. In rice, however, the same mutation does not affect embryo development but instead causes a lethal albino phenotype at the seedling stage. Interestingly, the *white leaf and panicle 3* (*wlp3*) mutation in the gene encoding uL18c (OsPRPL18) in rice does not result in lethality. Instead, the albino phenotype is restricted to the second leaf stage and persists until the tillering stage [31]. Furthermore, in rice, these mutations disturb plastid ribosome biogenesis and plastid intron splicing [103] and significantly reduce the expression levels of genes associated with plastid development [31].

The missense mutation (*cdm*, *chlorophyll-deficient mutant*) in the plastid genome-encoded uS4C (BrPRPS4) gene in Chinese cabbage (*Brassica rapa*) leads to significant changes in the quantitative leaf proteomics of proteins encoded by both plastid and nuclear genomes. These proteomic changes highlight differences in metabolic pathways between mutant and WT plants. A total of 233 proteins, associated with processes such as gene expression regulation (including methylation), plastid translation, ribosomes, and spliceosomes, are upregulated, while 307 proteins, primarily related to photosynthesis, are downregulated in the *cdm* phenotype [30]. Moreover, the *cdm* mutation disrupts the processing of rRNA that constitutes plastid ribosomes [127]. As a result, multiple plastid processes—particularly ribosome biogenesis and functions, and consequently plastid translation—are adversely affected. This disruption leads to chlorophyll deficiency and reduced photosynthetic efficiency, ultimately resulting in the slow growth of plants with yellow leaves [30].

### 4.2. Plastid RPs in Stress Response

*Abiotic stress*. Interestingly, plants exhibit sensitivity to temperature changes through the activity of certain PRPs. These proteins are crucial for proper plastid development at optimal temperatures and play a significant role in overall plant resistance, particularly against low-temperature stress. Their deficiency can induce chilling stress and cause cellular damage. In rice, the bS6c (OsPRPS6) protein, encoded by the nuclear gene *TCD11*, is highly expressed in leaves and is essential for chloroplast development, structure, and function. Its roles include organizing thylakoid membranes, regulating the transcription of chloroplast-related genes, and supporting chloroplast ribosome biogenesis, particularly at temperatures below 20 °C. The *tcd11* mutation impairs these processes, resulting in a lethal albino phenotype at the seedling stage under low-temperature conditions. However, at 32 °C, plants with the *tcd11* mutation exhibit a phenotype indistinguishable from that of WT plants [114].

The genes encoding uL3c (OsPRPL3) and uL13c (OsPRPL13) in rice are essential for chloroplast development and functioning. These genes are particularly highly expressed in green tissues, primarily in the leaves of young seedlings, and their protein products are localized to chloroplasts. Rice plants with mutations in these genes exhibit altered expression of genes involved in chloroplast development and related processes, such as transcription, translation, and photosynthesis. Consequently, these mutations result in lethal albino seedlings with defective chloroplasts from the proplastid stage [113,128]. Interestingly, the *wlp1* (*white leaf and punicles 1*) mutation in the gene encoding uL13c (OsPRPL13) in rice causes similar defects but only under low-temperature conditions. Whereas, at 30 °C, *wlp1* mutants show phenotypic similarity to WT plants. The *WLP1* gene is induced by low temperatures, indicating its critical role in proper chloroplast development in young rice seedlings under chilling conditions [129].

bL33C (NtPRPL33), encoded by the plastid genome, also plays a significant role in the functioning of tobacco *N. tabacum*) plants at low temperatures. Plants with a mutation in this gene exhibit reduced tolerance to low-temperature stress and prolonged recovery period due to cold-induced photooxidative damage, compared to WT plants. However, under optimal growth conditions, the plant can function normally without this protein, as it is not essential for translation, and ribosomes are able to function in its absence [105].

The above-mentioned uS5c (AtPRPS5) may also contribute to cold stress tolerance in *Arabidopsis*, as the *rps5* mutation reduces the abundance of cold stress response proteins. Mutant plants show decreased cold tolerance, whereas plants with overexpressing uS5c (AtPRPS5) exhibit enhanced cold tolerance and recover more efficiently from low-temperature stress [115]. Similarly, plants with the *cdm* mutation (*CLOROPHYLL-DEFICINT MUTANT*, which encodes uS4C [BrPRPS4] in Chinese cabbage) demonstrate increased sensitivity to low temperatures due to reduced expression of proteins involved in the cold stress response [30].

Some PRPs are involved in plant responses to high temperatures. The heat-responsive chloroplast bS1c (AtPRPS1) plays an essential role in retrograde activation of the heat stress response in *Arabidopsis*. Knockdown of the *bS1c* (*AtPRPS1*) gene inhibits the expression of the heat stress-activated *HsfA2* gene and its target genes, including HSPs, resulting in a heat-sensitive phenotype. Moreover, the mutant phenotype exhibits impaired biosynthesis of thylakoid membrane proteins encoded by the chloroplast genome, along with a lack of thylakoid stability, leading to pale green, size-reduced plants [32].

*Biotic stress*. Beyond their role in responses to abiotic stresses, some chloroplast proteins, including certain PRPs, are involved in responses to pathogen infections, including their recruitment by viruses to facilitate plant infection. In *N. benthamiana*, the chloroplast uL1c (NbPRPL1) protein acts as a host factor in tobacco vein banding mosaic virus (TVBMV) infection. The interaction between uL1c (NbPRPL1) and TVBMV RNA-dependent RNA polymerase (nuclear protein inclusion b, NIb) prevents Nib’s degradation via the NbBeclin-1 pathway. This interaction enhances potyviral replication and pathogenesis. Conversely, knocking out the gene encoding uL1c (NbPRPL1) reduces TVBMV replication and systemic transport, whereas its overexpression leads to increased accumulation of NIb and viral RNA [130].

### 4.3. Other Plastid RP-Related Functions

In addition to PRPs, other factors involved in plastid ribosome biogenesis play important roles in plant development. One such factor is the plastid ribosome assembly factor EMB15, encoded by the *EMBRIO DEFECTIVE 15* gene in the nuclear genome of maize. EMB15 contains a ribosomal maturation factor domain that facilitates interaction with uS19C (ZmPRPS19). Mutation of the gene encoding EMB15 disrupts its interaction with uS19C (ZmPRPS19) and impairs the maturation of plastid 16S rRNA. This disruption adversely affects the assembly of SSU, plastid translation, and overall plastid functions. Consequently, seed development is impacted, particularly delaying the early stages of embryo development without affecting the endosperm. Depending on the genetic background, the mutation can result in either an embryo-lethal phenotype or an albino phenotype [131].

Interestingly, quantitative analysis of PRPs can provide insights into specific plant characteristics. For example, it has been suggested that the abundance of these proteins, particularly their overexpression, may serve as an indicator of trait heterosis and hybrid vigor in adult hybrid plants. This hypothesis was supported by observations in maize hybrid seedlings, where increased expression of genes encoding chloroplast RPs, along with associated specific features, was noted [132].

The plastid/chloroplast RP functions presented in this paragraph are shortly summarized in Table 2.

## 5. Functions of Mitochondrial RPs (mitRPs)

Although mitochondrial ribosomes (mitoribosomes) originate from prokaryotes, they exhibit structural and compositional diversity compared to their procaryotic counterparts. Unlike prokaryotic, cytosolic, or plastid ribosomes, which are relatively uniform in their RP and RNA composition, mitochondrial ribosomes are unique in this respect due to their distinct rRNA/tRNA components. This specialization makes them highly adapted to the needs of mitochondrial translation. Moreover, mitoribosomes vary among different groups of eukaryotic organisms [133,134]. The protein composition of mitoribosomes can also change within a given species, for instance, during development, enabling them to perform specialized functions. Notably, plant mitoribosomes differ significantly from those in bacteria and other eukaryotes in terms of both structure and protein composition. For example, *Arabidopsis* mitoribosomes are among the largest and most complex known. Their sedimentation coefficient is 78S, which is attributed to the unusual size of SSU, larger than LSU [61]. This discrepancy arises from an additional rRNA domain grafted onto SSU’s head and the integration of numerous ribosomal pentatricopeptide repeat (rPPR) proteins [135]. The species-specific characteristics of mitoribosomes have been comprehensively documented in several excellent reviews [62,136,137,138].

The functioning of mitochondria is coordinated by both the mitochondrial and nuclear genomes. However, the proteins biosynthesized by mitochondrial ribosomes are based on the translation of transcripts encoded by mitochondrial genes. While all rRNAs that constitute mitoribosomes are encoded by the mitochondrial genome across all organisms, the situation differs for mitochondrial ribosomal proteins (mitRPs). In plants, only four mitRPs of the mitochondrial LSU and four mitRPs of SSU are encoded by the mitochondrial genome, with the remaining mitRPs encoded by the nuclear genome. In contrast, in animals, all mitRPs are encoded by the nuclear genome [62,139]. In *A. thaliana*, mitoribosomes consist of 94 mitRPs, including 49 associated with LSU and 44 with SSU (Figure 3) [61]. Among mitRPs, 19 are plant-specific, including 10 rPPR proteins and 1 mitRP of ambiguous provenance. These rPPR proteins play key roles in recruiting and anchoring mitochondrial mRNAs to SSUs and act in mitochondrial gene expression as generic translation factors in mitochondrial translation [61,135]. For example, mS76 (AtrPPR1) is believed to maintain optimal mitochondrial translation. A mutation of the gene encoding mS76 (AtrPPR1) significantly reduces the efficiency of mitochondrial mRNA translation, thereby limiting the production of proteins encoded by the mitochondrial genome. Generally, dysfunctions in genes encoding various rPPR proteins result in diverse phenotypes. Mutations in some rPPR genes can cause severe growth delay, impaired seed production, or even lethality. However, mutations in others, such as *mS78* (*AtrPPR3a)* or *mS81* (*AtrPPR8*), do not produce clear phenotypic differences compared to WTs [135].

The synthesis of mitRPs encoded by the mitochondrial genome is hypothesized to play a crucial role in regulating mitoribosome assembly [139]. RPs that are parts of mitochondrial ribosomes are primarily involved in the biosynthesis of respiratory chain proteins, thereby influencing processes essential for mitochondrion functioning. Since mitochondria are ubiquitous structures in eukaryotic cells, including those of plants, and are crucial for maintaining the cell’s and organism’s energetic status, they perform systemic functions. Therefore, mutations in genes encoding mitRPs can impair mitochondrial activity, leading to deficiencies in mitochondrial translation and, consequently, disruption of various biological processes. Such disruptions may result in severe developmental abnormalities at different stages, ranging from reproductive impairments to defects in vegetative tissue development and even lethal phenotypes [40]. Furthermore, it has been suggested that, beyond their role in mitochondrial translation, mitoribosomes may participate in regulating gene expression by forming expressosome-like structures. In these structures, mitoribosomes interact with factors involved in mitochondrial gene regulation and are, therefore, thought to control mitochondrial gene expression [61].

Mitochondrial ribosomes, like their cytosolic counterparts, exhibit heterogeneity due to the presence of mitRP paralogs with distinct functional roles. Twelve mitRPs are encoded by multiple genes, forming small gene families [6]. This diversity results in mitoribosome populations containing different mitRP variants, which translate mRNAs into proteins addressed to specific tissues and developmental stages. For instance, a mitoribosome variant containing one of the highly divergent members of the uL18m (AtMRPL18) family, encoded by *HEART STOPPER* (*HES*, At1g08845), is preferentially expressed in proliferating tissues of *Arabidopsis*, such as the embryo and root meristem [140].

*uS9m* (*AtMRPS9*) plays a crucial role in the development of male and female gametophytes, as well as seeds, in *A. thaliana*. Normally, *uS9m* (*AtMRPS9*) is highly expressed in both male and female gametophytes, both prior to and following double fertilization. Initial studies demonstrated that the *us9m* (a*tmrps9*) mutation results in defects in the female gametophyte. Although embryo sacs with the *us9m* (a*tmrps9*) mutation are successfully fertilized, early embryo stages and endosperm development are disturbed due to incorrect cell divisions [141,142]. The phenotype carrying the *us9m* (a*tmrps9*) mutation is similar to that observed in plants with mutations in *ANK6* (*ANKYRIN-REPEAT PROTEIN*), a mitochondrial protein required for fertilization. The interaction between uS9m (AtMRPS9) and ANK6 suggests that these proteins jointly regulate female gametophyte development, likely by controlling the expression of specific mitochondrial genes during this process [141]. Subsequent research revealed that *uS9m* (*AtMRPS9*) also plays a significant role in male gametogenesis. The *us9m* (a*tmrps9*) mutation leads to abnormal pollen development and defective pollen tube growth [142].

Other mitRPs genes also play key roles in reproductive processes. For example, *NUCLEAR FUSION DEFECTIVE 1* (*NFD1*), encoding bL21m (AtMRPL21), is essential for the maturation of the central cell and endosperm in *Arabidopsis* [143]. Similarly, *GAMETE CELL DEFECTIVE 1* (*GCD1*), putatively encoding bL20m (AtMRPL20), is critical for these processes as well [144]. Similarly, a mutation in the *HES* gene, encoding uL18m (AtMRPL18), results in inhibited embryo development, uncellularized endosperm, and impaired seed development in *Arabidopsis* [140].

Reproductive processes require high energy input, and their disruption in plants with mutated genes encoding mitRPs, including the *uS9m* (*AtMRPS9*) gene, is likely due to impaired biosynthesis of mitochondrial genome-encoded proteins, including those involved in energy production. This dysfunction stems from defects in mitochondrial ribosome biogenesis. Furthermore, these processes are further compromised by ROS production in plants with mutations in mitRPs [141,142].

*DEK44* (*DEFECTIVE KERNEL 44*) is a conserved gene in monocots that encodes the bL9m (ZmMRPL9) protein in maize. Although this gene is widely expressed across most tissues at the transcript level, its protein product accumulates exclusively in the kernels. The DEK44 protein plays a crucial role in regulating kernel growth and development, including the embryo and endosperm, by influencing factors that control the cell cycle. In the *dek44* mutant, significant reduction in cell proliferation, delayed endosperm development, and embryo lethality are observed. As a result, the kernels are small and incapable of germinating. Furthermore, the loss of *DEK44* function disrupts the expression of respiratory chain proteins encoded by both mitochondrial and nuclear genomes. This leads to significantly impaired assembly of respiratory chain complexes, along with defects in mitochondrial biogenesis, morphology, and function. Interestingly, in mutants with a dysfunctional gene encoding bL9m (ZmMRPL9), an upregulation of other electron transport chain proteins is observed. This may represent a compensatory response to the perturbed mitochondrial translation of specific respiratory chain proteins. It is hypothesized that DEK44 functions as a regulatory filter for the biosynthesis of specific proteins from selected mRNAs in the mitochondria of maize kernel cells [145].

The nuclear gene encoding mitochondrial uS10m (AtMRPS10) is essential for the proper development of *A. thaliana*. Transgenic plants with reduced levels of uS10m (AtMRPS10) transcripts exhibit severe developmental defects, including various morphological abnormalities or even lethality during the vegetative growth phase, compared to WT plants. These phenotypic changes depend on factors such as homozygous or hemizygous gene silencing, the timing of silencing onset, developmental stage, and environmental conditions [146]. The disturbed development observed in plants deficient in uS10m (AtMRPS10) may stem from alterations in mitochondrial translation. Notably, differences between the mitochondrial transcriptome and translatome have been identified in the *us10m* (*atmrps10*) mutant. In plants with silenced *uS10m* (*AtMRPS10*), some mitochondrion-encoded mitRPs were over-synthesized, while certain protein components of the oxidative phosphorylation apparatus were downregulated. Interestingly, transcripts encoding these proteins were upregulated in both systems, whereas transcript levels of other nuclear-encoded components of these systems were largely unaffected. This suggests that mitochondrial ribosomes might play a regulatory role in mitochondrial translation, selectively guiding the process [147]. Moreover, uS10m (AtMRPS10) is believed to be involved in mitochondrial splicing, as this process was found to be less efficient in the *us10m* (*atmrps10*) mutant. The observed connections between uS10m (AtMRPS10) and transcription, splicing, and translation highlight its role in the regulation of mitochondrial gene expression [24].

As noted above, some members of a given RP family perform typical ribosomal functions, while others may take on extraribosomal roles. In *Arabidopsis*, eight nuclear-encoded members of the uL18 family are directed to both mitochondria and chloroplasts. Among them, uL18L1 and uL18L8, which are not components of ribosomes, are involved in the splicing of specific introns of pre-mRNAs transcribed in mitochondria and chloroplasts, respectively. Defective splicing caused by mutations in the genes encoding these homologs negatively impacts organelle functionality and overall plant development, resulting in abnormal phenotypes. Plants with the *ul18l1* mutation exhibit significantly slower growth, twisted leaves, delayed flowering, and seeds with reduced germination capacity. Additionally, these mutants show a reduction in respiratory complex I, underscoring the critical role of uL18L1 in maintaining the mitochondrial activity of this complex. In contrast, the *ul18l8* mutation results in less severe defects than *ul18l1*. These phenotypes include a moderate reduction in photosynthetic efficiency, slightly lower chlorophyll and carotenoid levels, and only marginally slower growth. Interestingly, both uL18L1 and uL18L8 possess domains similar to those involved in binding 5S rRNA. It is hypothesized that these proteins may have indirect ribosomal functions by interacting with 5S rRNA, thereby promoting its incorporation into the 50S ribosomal subunits in mitochondria and plastids [90].

The functions of mitochondrial RPs discussed in the section above are summarized shortly in Table 3.

## 6. Conclusions

Numerous lines of evidence suggest that proteins, including RPs, and even RNAs, which have traditionally been assigned specific roles, may also perform additional functions that influence a wide range of cellular processes. These processes can be regulated at various levels, depending on the physiological state, developmental stage, or environmental conditions.

RPs, as key ribosome building blocks, are essential for translational activity but may also be engaged in extraribosomal roles. In plants, the widespread duplication of RP genes may partially explain the functional diversity observed within RP families. This diversity among RP paralogs likely contributes to ribosome heterogeneity, enabling the formation of specialized ribosomes capable of selectively translating specific mRNAs. At the same time, some RPs may have entirely non-ribosomal functions. In both cases, RPs can influence morphogenetic and physiological processes or responses to various environmental stimuli. Consequently, deficiencies in RPs can compromise ribosome biosynthesis, functionality, or availability, thereby disrupting protein biosynthesis. Simultaneously, RP scarcity may impact their non-translational roles, such as the regulation of specific cellular processes, acting as signaling molecules, or functioning as intermediaries in metabolic networks. These disruptions can lead to abnormalities in cellular processes and result in phenotypic deviations from WT plants.

Mutational defects in RPs often lead to diverse outcomes depending on the particular RP affected. Moreover, a single RP can participate in multiple processes, making it challenging to attribute specific functions to its corresponding gene based on a single mutation. Hence, the functions of individual RPs, including their extraribosomal roles, have been elucidated for only a small subset of these proteins. Furthermore, different mutations in the same RP gene can result in distinct phenotypes. Most studies investigating phenotypic changes and functional roles of specific RPs rely on natural or induced mutations that suppress or alter the expression of the corresponding genes. Although advances in molecular and biotechnological approaches have significantly expanded our understanding of individual RP functions, it remains unclear whether the observed phenotypes stem from the translational roles of RPs within ribosomes or from their non-ribosomal functions. To fully uncover and understand the surprising and novel roles of RP-encoding genes, comprehensive functional analyses are required. This would involve constructing systems with diverse mutations in a given gene and examining their effects on morphology and physiological processes throughout the plant’s life cycle and under various environmental conditions. Each RP should, therefore, be considered a multifaceted molecule with potential functions extending far beyond translation.

Understanding the mechanisms and factors that enable RPs to participate in these diverse roles holds great potential for practical applications. Insights gained from such research could, for example, help in developing plants better adapted to fluctuating and unfavorable environmental conditions, ultimately improving crop yields and resilience.

## Figures and Tables

**Table 1:** Brief description of the functions of plant cytosolic ribosomal proteins (RPs) and the processes they influence, as discussed in the article.

Cytosolic RP		Species	Function/Process	References
Name	Old Name			
uL1y	RPL10aB	*A. thaliana*	Leaf development	[25]
uL4y	RPL4D	*A. thaliana*	Leaf development, lipid biosynthesis	[25,89]
eL6	RPL6	*O. sativa*	Abiotic stress response (osmotic, drought, salt stress)	[21,35,71]
uL6	RPL9	*A. thaliana*	Chilling stress, cold acclimation	[73]
uL11y	RPL12B	*A. thaliana*	Abiotic stress response (Pi deficiency)	[34]
eL14y	RPL14B	*A. thaliana*	Fertilization, embryogenesis, pollen tube development and functionality	[63]
eL15	RPL15	*O. sativa*	Biotic stress response (defense against BPH and gall midge)	[37]
uL15	RPL27a	*A. thaliana*	Male and female gametophyte development, pollen grain, and embryo sac development	[64]
uL16 uL16z	RPL10 RPL10A	*A. thaliana* *N. benthamiana* *A. thaliana*	Abiotic stress response (UV-B stress); Biotic stress response (defense against bacterial host and non-host–pathogens); Early stages of plant development	[36,80] [23]
eL18x	RPL18C	*A. thaliana*	Leaf development	[25]
uL18L1/ uL18L8		*A thaliana*	Splicing and LSU biogenesis in mitochondria/plastid, overall plant development	[90]
eL20y eL20	RPL18aB RPL18a	*A. thaliana* *O. sativa*	Early embryogenesis, seed development, male gametophyte functioning, suspensor development; Biotic stress response (defense against gall midge)	[65,66] [37]
uL22	RPL22	*O. sativa*	Biotic stress response (defense against gall midge)	[37]
uL23z uL23z/y	RPL23A RPL23aA/aB	*O. sativa* *A. thaliana*	Abiotic stress response (osmotic, drought, salt stress); overall plant development;	[21,35,71] [85,86]
eL24z	RPL24A	*A. thaliana*	Abiotic stress response (osmotic stress)	[67]
eL28z	RPL28A	*A. thaliana*	Leaf development	[25]
uL30y	RPL7B	*A. thaliana*	Leaf development	[25]
eL32 eL32z	RPL32 RPL32A	*O. sativa*	Response to various abiotic stresses; cold, salt, drought, sucrose stress	[44]
eL36	RPL36.2	*O. sativa*	Biotic stress response (defense against gall midge)	[37]
eL37	RPL37	*G. max*	Cold stress response	[72]
eL38 eL38y	RPL38 RPL38B	*O. sativa* *A thaliana*	Biotic stress response (defense against gall midge); leaf development	[37] [25]
eL39x	RPL39C	*A thaliana*	Leaf development	[25]
eS1z	RPS3A	*O. sativa*	Leaf development	[84]
uS4	RPS9.2	*O. sativa*	Biotic stress response (defense against gall midge)	[37]
es6 eS6z	RPS6 RPS6A	*G. max* *N. benthamiana* *G. hirsutum* *A. thaliana*	Abiotic (cold) cold stress response; Biotic stress response (response to viruses); Positive biotic stress response (defense against *V. dahliae*); rDNA transcription regulation; Leaf development;	[72] [75,76,77] [78] [88] [25]
uS7y/z	RPS5/a	*O. sativa*	Biotic stress response (insect resistance to BPH and gall midge)	[37]
eS10y	RPS10B	*A. thaliana*	Shoot meristem functioning	[87]
us15	RPS13	*G. max*	Cold stress response	[72]
eS21 eS21z	RPS21 RPS21B	*C. sativus* *A. thaliana*	Positive biotic stress response (defense against CCYV); Leaf development	[74] [25]
eS24y	RPS24B	*A. thaliana*	Leaf development	[25]
eS25	RPS25a	*O. sativa*	Biotic stress response (defense against gall midge)	[37]
eS28y eS28z	RPS28B RPS28A	*A. thaliana*	Leaf development	[25]

**Table 2:** Brief description of the functions of plastid/chloroplast ribosomal proteins (RPs) and the processes they influence, as discussed in the article.

Plastid/Chloroplast RP		Species	Function/Process	References
Name	Old Name			
uL1c	RPL1	*A. thaliana* *N. benthamiana*	Early stages of embryo development; Biotic stress response (promotes infection by TVBMV)	[27] [130]
uL3c	RPL3	*O. sativa*	Chloroplast development and functioning	[129]
uL4c	RPL4	*A. thaliana*	Early stages of embryo development	[27]
uL5c	RPL5	*A. thaliana*	Early stages of embryo development	[106]
bL12c	RPL12	*O. sativa*	Chloroplast development and photosynthesis at early stages of seedling growth	[119]
uL13c	RPL13	*O. sativa*	Chloroplast development and functioning, particularly under low temperatures	[129]
uL18c	RPL18	*A. thaliana*, *O. sativa*	Chloroplast development and functioning, chlorophyll biosynthesis, early embryo/seedling stage development	[31]
bL20	RPL20	*N. tabacum*	Photosynthesis, plant development, and survival	[104,105]
bL21c	RPL21	*O. sativa*	Chloroplast development and photosynthesis at early stages of seedling growth	[29]
uL24c	RPL24	*A. thaliana*	Photosynthesis	[27]
bL27c	RPL27	*A. thaliana*	Early stages of embryo development	[27]
bL28c	RPL28	*A. thaliana*	Later stages of embryo-to-seedling development	[27]
bL33C	RPL33	*O. sativa*	Abiotic stress response (plant functioning under low temperatures)	[105]
bL35	RPL35	*A. thaliana*	Early stages of embryo development	[27]
cL37	PSRP	*A. thaliana*	Photosynthesis, chloroplast development, plastid rRNA processing, leaf variegation	[91,116]
bS1c	RPS1	*A. thaliana* *O. sativa*	Photosynthesis; Abiotic stress response (heat stress); Retrograde signaling	[27] [32] [126]
uS2C	RPS2	*N. tabacum*	Photosynthesis, plant development, and survival	[104,105]
uS4C	RPS4	*N. tabacum* *C. cabbage*	Photosynthesis, plant development and survival; Abiotic stress response (cold stress tolerance)	[104,105] [30]
uS5c	RPS5	*A. thaliana*	Photosynthesis, overall plant development; Abiotic stress response (cold stress tolerance)	[115] [30]
bS6c	RPS6	*O. sativa*	Chloroplast development and functioning under low temperatures	[114]
uS9c	RPS9	*A. thaliana*	Chloroplast development, photosynthesis, plastid rRNA processing, leaf variegation	[116]
uS11C	RPS11	*A. thaliana*	Plastid rRNA processing	[116]
uS17c	RPS17	*A. thaliana* *Z. mays*	Photosynthesis	[27] [112]
bS18C	RPS18	*N. tabacum*	Photosynthesis, plant development, and survival	[104,105]
uS19C	RPS19	*Z. mays*	Plastid 16 rRNA maturation	[131]
bS20c	RPS20	*A. thaliana* *O. sativa*	Early stages of embryo development; Chloroplast development and photosynthesis at early stages of seedling growth; Retrograde signaling	[27] [120] [126]
bS21c	RPS21	*A. thaliana*	Photosynthesis, carbon/nitrogen balance regulation, overall plant development; Sensitivity to glucose excess, fertilization	[117] [118]
cS22	PSRP2	*A. thaliana*	Negative regulation of seed germination under abiotic stresses (salinity, dehydration, low temperature)	[107]
cS23	PSRP3	*A. thaliana*	Photosynthesis	[91]
bTHXc	PSRP4	*A. thaliana*	Photosynthesis	[91]

**Table 3:** Brief description of the functions of plant mitochondrial ribosomal proteins (RPs) and the processes they influence, as discussed in the article.

Mitochondrial RP		Species	Function/Process	References
Name	Old Name			
bL9m	RPL9	*Z. mays*	Embryo, endosperm, kernel development	[145]
uL18m	RPL18	*A. thaliana*	Proliferating tissues functioning (embryo and root meristem)	[140]
bL20m	RPL20	*A. thaliana*	Central cell and endosperm maturation; Embryo, endosperm, seed development	[144] [140]
bL21m	RPL21	*A. thaliana*	Central cell and endosperm maturation	[143]
uS9m	RPS9	*A. thaliana*	Reproductive processes (male and female gametogenesis)	[141,142]
uS10m	RPS10	*A. thaliana*	Plant development during the vegetative phase; Mitochondrial gene expression (transcription, splicing, translation)	[146] [24,147]

## Data Availability

No new data were created or analyzed in this study.
